# Precision diabetes: learning from monogenic diabetes

**DOI:** 10.1007/s00125-017-4226-2

**Published:** 2017-03-17

**Authors:** Andrew T. Hattersley, Kashyap A. Patel

**Affiliations:** The Institute of Biomedical and Clinical Science, University of Exeter Medical School, RILD Building, Level 3, Royal Devon and Exeter Hospital, Barrack Road, Exeter, EX2 5DW UK

**Keywords:** GCK, HNF1A, HNF4A, KCNJ11, Maturity onset diabetes of the young, MODY, Monogenic diabetes, Neonatal diabetes, Precision diabetes, Precision medicine, Review, Type 2 diabetes

## Abstract

The precision medicine approach of tailoring treatment to the individual characteristics of each patient or subgroup has been a great success in monogenic diabetes subtypes, MODY and neonatal diabetes. This review examines what has led to the success of a precision medicine approach in monogenic diabetes (precision diabetes) and outlines possible implications for type 2 diabetes. For monogenic diabetes, the molecular genetics can define discrete aetiological subtypes that have profound implications on diabetes treatment and can predict future development of associated clinical features, allowing early preventative or supportive treatment. In contrast, type 2 diabetes has overlapping polygenic susceptibility and underlying aetiologies, making it difficult to define discrete clinical subtypes with a dramatic implication for treatment. The implementation of precision medicine in neonatal diabetes was simple and rapid as it was based on single clinical criteria (diagnosed <6 months of age). In contrast, in MODY it was more complex and slow because of the lack of single criteria to identify patients, but it was greatly assisted by the development of a diagnostic probability calculator and associated smartphone app. Experience in monogenic diabetes suggests that successful adoption of a precision diabetes approach in type 2 diabetes will require simple, quick, easily accessible stratification that is based on a combination of routine clinical data, rather than relying on newer technologies. Analysing existing clinical data from routine clinical practice and trials may provide early success for precision medicine in type 2 diabetes.

**Electronic supplementary material:**

The online version of this article (doi:10.1007/s00125-017-4226-2) contains a slideset of the figures for download, which is available to authorised users.

## Introduction

Precision medicine refers to the tailoring of medical treatment to the individual characteristics of each patient or subpopulation [1]. Precision diabetes is when a precision medicine approach is used to improve treatment of patients with diabetes. This review aims to examine how precision diabetes has been successfully applied in monogenic diabetes and to ask what this can teach us about the challenges of implementing a precision diabetes approach in type 2 diabetes. For a detailed discussion of precision diabetes for type 2 diabetes, please see review by Mark McCarthy in this issue of *Diabetologia* [2].

## Overview of the precision medicine approach in monogenic diabetes

### Overview of neonatal diabetes

#### Genetic causes of neonatal diabetes

Neonatal diabetes is defined as diabetes developed before 6 months of age. The knowledge that neonatal diabetes has a monogenic aetiology is based on two strong strands of evidence; first, patients with permanent diabetes diagnosed <6 months of age do not have an increased type 1 diabetes genetic susceptibility. This is in contrast with the high susceptibility seen when those diagnosed >6 months [3, 4]. Second, 96% of patients with known ‘neonatal’ monogenic genetic aetiology are diagnosed with diabetes < 6 months [5, 6]. Before genetic definition was possible, neonatal diabetes was classified solely on the clinical course of disease as transient neonatal diabetes (TNDM), permanent neonatal diabetes (PNDM), or by the specific syndrome when associated with other features e.g. Wolcott–Rallison syndrome or immunodysregulation polyendocrinopathy enteropathy X-linked (IPEX) syndrome. We now know of 23 different genetic causes of neonatal diabetes [7, 8].

#### Genetic diagnosis: impact on diabetes treatment

**Fig. 1:**
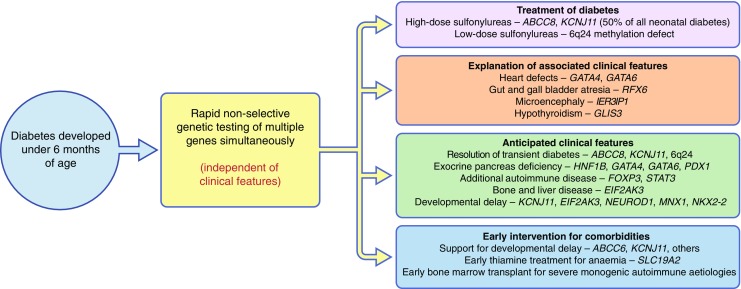
The paradigm shift: early non-selective genetic testing for neonatal diabetes. The figure shows the benefits of early comprehensive genetic testing in the management of neonatal diabetes. For example, genotype may: (1) predict treatment response: e.g. patients with mutations in *KCNJ11*/*ABCC8* respond well to high-dose sulfonylureas; (2) explain pre-existing clinical features (e.g. heart defects with *GATA4/6* mutation, microencephaly with *IER3IP1* mutation and hypothyroidism with *GLIS3* mutation); (3) shed light on anticipated clinical abnormalities (e.g. exocrine pancreas deficiency with mutations in *GATA4*, *GATA6* or *PDX1*, and bone and liver disease with *EIF2AK3* mutations); (4) lead to early intervention for co-morbidities, such as early treatment with high doses of sulfonylureas in *KCNJ11*-neonatal diabetes to improve the outcome when the mutation causes severe developmental delay, or early treatment with thiamine in thiamine-responsive megaloblastic anaemia (TRMA) neonatal diabetes

The key drive for precision diabetes has been the finding that the genetic aetiology strongly influences treatment choice and the clinical course (Fig. 1) [7]. Approximately 50% of patients with neonatal diabetes have mutations in the genes encoding the potassium channel (*KCNJ11*, *ABCC8*). These patients show excellent glucose control with high-dose sulfonylureas without an increase in hypoglycaemia and glucose variability [9]. They also show improvements in their neurological function following sulfonylurea therapy [10, 11]. Patients with transient neonatal diabetes as a result of 6q24 methylation abnormalities can be treated with low-dose sulfonylureas when they relapse (Fig. 1). In contrast, patients with other neonatal diabetes subtypes require insulin treatment.

#### Genetic diagnosis: impact on clinical course

Another key benefit of precision medicine is the ability to explain additional clinical abnormalities that are associated with the underlying genetic cause (Fig. 1). These may be already present (e.g. cardiac defects in patients with *GATA6* mutations, microencephaly in patients with *IER3IP1* mutations and gall bladder and gut atresia in patients with *RFX6* mutations), anticipated (e.g. exocrine pancreas deficiency in patients with mutations in *GATA4*, *GATA6* or *PDX1*, and remission of transient diabetes in patients with 6q24 methylation abnormalities) or they may develop later (e.g. hepatic failure and bone abnormalities in Wolcott–Rallison syndrome or other autoimmune conditions with IPEX syndrome) [7].

#### Early comprehensive genetic testing: a paradigm shift for managing neonatal diabetes

The development in targeted next-generation DNA sequencing has allowed rapid and comprehensive testing of all known genetic aetiology in monogenic diabetes [12]. In parallel, referral time from development of diabetes to genetic testing reduced from 4 years to 7 weeks between 2004 and 2013 [7]. These two factors have led to a paradigm shift in the way that we manage neonatal diabetes as we can now make a rapid and precise genetic diagnosis before the development of all clinical features (Fig. 1). This can lead to early appropriate treatment of the diabetes and future planning for other clinical developments [7]. For example, early diagnosis of TNDM allows remission to be predicted and planned for, and knowing that developmental delay is a feature of the genetic aetiology allows early developmental assessment and support. Furthermore, early treatment with sulfonylureas in *KCNJ11*- and *ABCC8*-neonatal diabetes probably results in less severe developmental delay [11]. In addition, early treatment with thiamine in thiamine-responsive megaloblastic anaemia (TRMA) neonatal diabetes can improve glycaemic control [13]. Finally, for IPEX and other severe monogenic autoimmune syndromes, early diagnosis allows consideration for early curative bone marrow transplantation before patients are too sick [14].

### Overview of MODY

#### Genetic causes of MODY

MODY was originally defined as a clinical subgroup of familial diabetes that was diagnosed early (typically before 25 years of age) but despite this, this condition was not insulin dependent and showed autosomal dominant inheritance [15, 16]. The initial linkage analysis in large families led to discovery of the first MODY gene, encoding glucokinase (*GCK*) [17, 18]. This was rapidly followed by discovery of genes encoding hepatic nuclear factor 1 alpha (*HNF1A*) [19], hepatic nuclear factor 4 alpha (*HNF4A*) [20] and hepatic nuclear factor 1 beta (*HNF1B*) [21]. Although other genetic causes have subsequently been described, none of these are as common as the four genetic causes initially described [22]. MODY represents between 1.2% and 3.0% of diabetes diagnosed in children, at least in predominately white European populations ([11, 12] and reviewed in [23]).

#### Discrete clinical features of common MODY subtypes

**Fig. 2:**
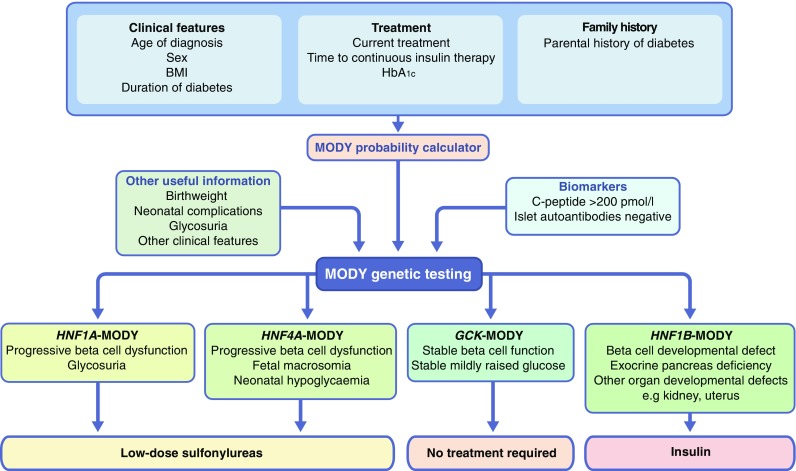
Identification, important clinical features and treatment implications for common subtypes of MODY. An individual’s clinical features, treatment needs and parental history of diabetes may be suggestive of MODY. A MODY probability calculator can use these features to predict probability of disease, and this calculation of MODY, plus biomarkers of disease and other useful clinical information will determine if MODY genetic testing should be carried out. Genetic testing allows for stratification of patients into specific MODY subgroups based on their genotype, which may be used to identify present and predicted clinical features and treatment responses. For example, patients with *GCK*-MODY have a stable, raised fasting glucose, whereas those with *HNF1A*-, *HNF4A*- or *HNF1B*-MODY experience progressive deterioration of glucose over time. Furthermore, glycosuria is a known feature of *HNF1A*-MODY, whilst fetal macrosomia and neonatal hypoglycaemia often occur in *HNF4A*-MODY, and developmental disorders of the kidney and multiple other organs in *HNF1B*-MODY

The discovery of the MODY genes led to the description of discrete clinical courses for different genetic subtypes (Fig. 2). *GCK*-MODY shows a stable, raised fasting glucose in contrast with the progressive deterioration of glucose over time observed with transcription factor-linked MODY (*HNF1A*-, *HNF4A*- and *HNF1B*-MODY; Fig. 2) [6, 24]. Patients with transcription factor-linked MODY have different associated features based on their underlying aetiology, such as glycosuria in *HNF1A*-MODY, fetal macrosomia and neonatal hypoglycaemia in *HNF4A*-MODY, and developmental disorders of the kidney and multiple other organs in *HNF1B*-MODY (Fig. 2) [6]. The majority of genetic testing requires initial clinical selection followed by molecular genetic testing of either the most likely gene or a panel of all MODY genes [12].

#### Differential treatment response in MODY subtypes

Probably the most important clinical feature associated with precision diabetes in MODY patients has been the differential treatment response in discrete genetic groups (Fig. 2). *GCK*-MODY patients do not require treatment [24, 25] and do not respond to either oral agents or low-dose insulin [24, 26]. In contrast, *HNF1A*- and *HNF4A*-MODY patients can be treated with low-dose sulfonylureas [6, 27, 28]. Patients who require additional treatment can have dipeptidyl peptidase-4 (DPP-4) inhibitors, glucagon-like peptide-1 (GLP-1) receptor agonist and insulin in addition to sulfonylureas. Patients with *HNF1B*-MODY require insulin treatment as the response to sulfonylureas and other oral medication is limited [29].

#### Increasing genetic diagnosis of MODY throughout the world

MODY genetic testing is increasing throughout the world and most developed countries have at least one academic, health service or commercial laboratory providing monogenic diabetes testing. Within the UK, the Exeter laboratory have gone from ∼50 patients being diagnosed with MODY in 1996 to ∼5000 diagnoses in 2016.

## Why has precision medicine in monogenic diabetes worked well?

### Subgroups that are clearly defined by underlying aetiology

**Fig. 3:**
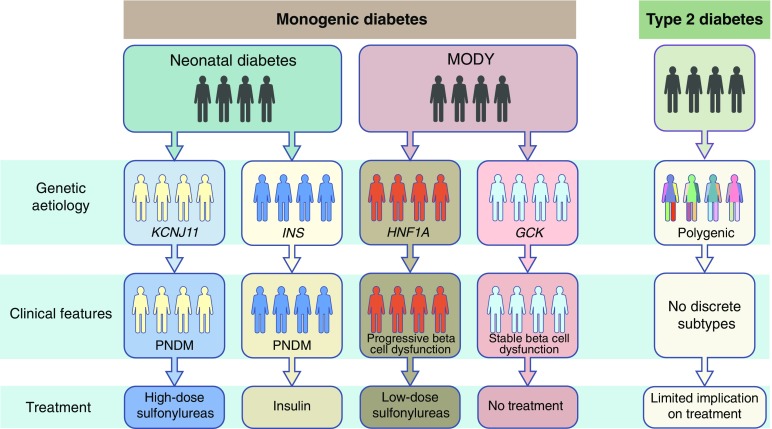
Molecular genetics-based approach for precision diabetes in monogenic and type 2 diabetes. An example of a precision diabetes approach in monogenic diabetes, in which molecular genetic aetiology defines subgroups that have differential features and treatment implications, is shown. A similar approach of precision diabetes in polygenic complex type 2 diabetes has failed to identify clear discrete aetiological subgroups and associated clinically useful treatment implications

A key feature of monogenic diabetes is that finding a mutation results in diagnosis of a specific subgroup. These specific subcategories have the advantage of being non-overlapping with differential clinical implications (Fig. 3). In addition, for many monogenic diabetes subtypes, the specific mutation will determine the clinical outcome because of genotype–phenotype relationships. For example, in *KCNJ11*-neonatal diabetes, the mutation severity determines the phenotype [30]. The functional impact of the mutation increases as the phenotype changes from TNDM to isolated PNDM, to PNDM with a neurological phenotype (developmental delay, epilepsy and neonatal diabetes [DEND]) [30, 31]. As another example, *HNF4A*-MODY patients with the p.R114W mutation (found in ∼15% of *HNF4A*-MODY patients) have different phenotypes compared with other *HNF4A*-MODY patients. They showed reduced sensitivity to low-dose sulfonylurea treatment, reduced penetrance and no effect on birthweight [32]. In contrast, *GCK-*MODY is interesting in that it has a uniform phenotype despite there being considerable functional differences in mutation severity [26]; this is due to compensation by overexpression of the normal allele [33].

### Large differences in treatment response in monogenic diabetes

Differences in treatment response can have a large impact in monogenic diabetes. The best example is the enhanced sensitivity to sulfonylureas in *HNF1A*-MODY, meaning that patients may become severely hypoglycaemic if standard doses are used, and that discontinuing sulfonylureas results in a marked deterioration in blood glucose (a 5% point reduction [31 mmol/mol] in HbA_1c_) [6, 34]. In a randomised trial sulfonylureas led to a fourfold greater reduction of fasting blood glucose in *HNF1A*-MODY patients compared with age, BMI and blood glucose level-matched type 2 diabetes patients [27]. This sensitivity to sulfonylureas was initially identified from clinical observation and was not predicted from gene function [34]. In contrast, there is a lack of glycaemic response with oral hypoglycaemic agents or low-dose insulin in patients with *GCK*-MODY [26]. The lack of efficacy of insulin treatment at a median dose of 0.4 U kg^−1^ day^−1^ is also seen in pregnancy as the birthweight of offspring of *GCK-*MODY patients treated with insulin and without insulin are similar [35]. Insulin is still recommended for individuals with *GCK-*MODY in some circumstances in pregnancy but even at very high doses, its ability to lower the mother’s blood glucose levels is limited (reviewed in [24]). The lack of response to therapy may be predicted because *GCK*-MODY patients have a regulated blood glucose that is set at a higher level, as a result of insulin and counter-regulatory hormones being regulated to maintain this elevated glucose level [24, 36].

High-dose sulfonylurea treatment in potassium channel-linked neonatal diabetes (*ABCC8*- and *KCNJ11*-neonatal diabetes) had a massive impact on endogenous insulin secretion (measured by C-peptide), which increased from an undetectable level to the level necessary to maintain glucose at near normal values [8]. This resulted in an ∼2 percentage point (22 mmol/mol) improvement in HbA_1c_ in the short term, which persisted for more than 5 years [9, 37]. Importantly, the basis for attempting this therapy arose from the knowledge that the potassium channel is the target of sulfonylureas.

## The difficulties of bringing a precision diabetes approach into monogenic diabetes care

### Neonatal diabetes: a success story of rapid implementation

The easy clinical recognition of neonatal diabetes combined with a dramatic treatment response following precise genetic diagnosis led to international guidelines being changed within 2 years after gene discovery [38]. The simple clinical guidance was issued that diagnostic genetic testing is required for all patients who developed diabetes before 6 months of age. The simplicity of this guidance greatly helped towards its rapid dissemination worldwide. This was further helped by support from the Wellcome Trust, allowing the Exeter Molecular Genetics Laboratory to offer free rapid comprehensive genetic testing throughout the world for patients with neonatal diabetes until at least 2020. This has resulted in over 1700 patients from 87 countries being tested for neonatal diabetes [7].

### MODY: slow uptake into clinical practice

**Fig. 4:**
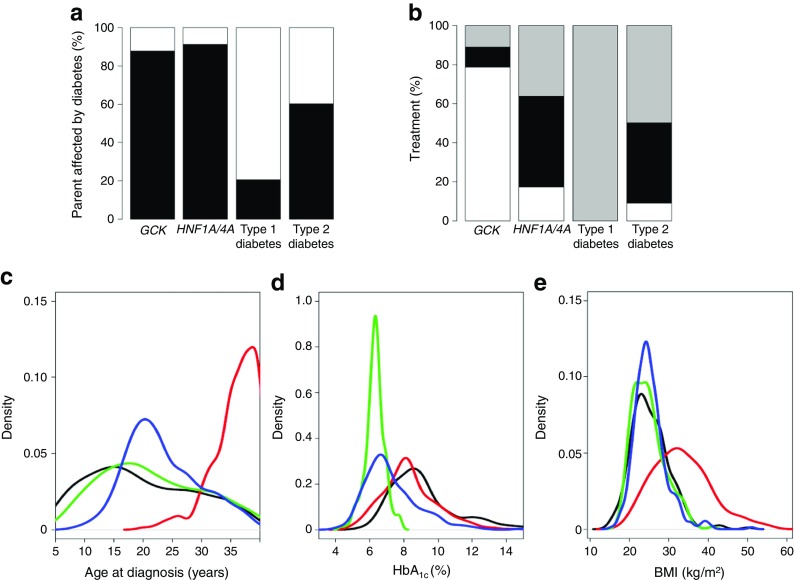
Clinical features of patients with MODY overlap with type 1 and type 2 diabetes. Percentage of (**a**) parent affected by diabetes (in black) and (**b**) treatment (diet, white; oral blood glucose lowering agents (OHA), black; insulin [± OHA], grey). Density plots for (**c**) age at diagnosis, (**d**) HbA_1c_ and (**e**) BMI (with child values converted to adult equivalent using reference charts [39]). For (**c**), (**d**) and (**e**), distributions for the four subtypes of diabetes are shown as: type 1 diabetes, black line; type 2 diabetes, red line; *GCK-*MODY, green line; *HNF1A*-/*HNF4A*-MODY, blue line. To convert values for HbA_1c_ in % into mmol/mol, subtract 2.15 and multiply by 10.929. All graphs are adopted from Shields et al [39] under the Creative Commons Attribution 4.0 International (CC BY) license (https://creativecommons.org/licenses/by/4.0) and amended to include colour

The recognition of MODY has been slow despite MODY being relatively common, the technology for genetic diagnosis being available in most countries and clear treatment recommendations having been defined once a diagnosis is made, both outside and during pregnancy [6, 24]. This is partly because precision diabetes based on genetic testing is a new concept for diabetologists; genetics is not a part of routine clinical training and, traditionally, the speciality of diabetes emphases are based on treatment rather than diagnosis. In addition, a major barrier for the dissemination of precision diabetes in MODY was the lack of single clinical criteria that can accurately identify all MODY patients. MODY cases overlap with type 1 and type 2 diabetes patients with regard to age of onset, BMI, history of parental diabetes, HbA_1c_ levels and treatment (Fig. 4) [39]. The traditional criteria of MODY (diabetes diagnosis <25 years, non-insulin treated and an affected parent) identify only 48% of MODY cases and, hence, are not sufficiently sensitive to be used alone in clinical practice [40]. The net result of these barriers is that the majority of MODY patients are not recognised [40, 41]. In addition, the cost of the genetic test is an important barrier to implementation, although early health economic evidence supports the cost effectiveness of genetic testing for both neonatal diabetes and MODY [42, 43].

### A solution to diagnosing MODY when there is no single criterion or threshold

Diagnosing MODY requires a complex multi-dimensional assessment of probability based on more than one clinical criterion. This may be difficult for clinicians but can be easily done by use of a statistical calculator that uses multiple, but readily available, clinical information to assess the probability of a patient having MODY. The ‘MODY Probability Calculator’ was developed by B. Shields and is available without charge at www.diabetesgenes.org and on the ‘Diabetes Diagnostic’ app for iOS and Android mobile platforms [39]. In a head-to-head competition, it proved to be as good as clinical experts with more than two decades of experience working with MODY (B. Shields, [University of Exeter Medical School, Exeter, UK] and A. T. Hattersley, personal communication). The probability calculator works best for patients who are not insulin treated. For patients who are insulin treated in whom the diagnosis of MODY is being considered, additional non-genetic tests (islet autoantibody testing and C-peptide analysis) should be considered as ‘rule-out tests’; the presence of islet autoantibodies and/or C-peptide <200 pmol/l effectively rules out MODY [44, 45].

The development of a MODY probability calculator has proved a very promising first step towards precision diabetes. The mobile app is widely used (>6000 downloads to date). This provides a good example of how sophisticated modelling of a complex diagnostic challenge can be simplified into a simple tool that uses readily available clinical information. This approach can greatly help rapid dissemination of precision diabetes.

### New technology does not make clinical selection redundant

Next-generation sequencing has transformed our ability to perform genetic testing but it has not removed the need for clinical selection of patients with possible monogenic diabetes for the genetic test. It is now possible to test all genes involved in monogenic diabetes in a single gene panel test, both quickly and efficiently [7, 12]. This gene panel testing approach identifies approximately an additional 25% of monogenic patients with less common causes compared with selected testing of common genetic subtypes [23]. It removes the need to define the likely genetic aetiology/subgroups prior to testing; however, extra care is needed when patients are not selected on phenotype as the prior likelihood of monogenic diabetes is greatly reduced and so false positive findings become more likely [46]. The easy access to sequencing technology has led to laboratories (including commercial laboratories) offering diagnostic testing for monogenic diabetes even when they do not have experience of monogenic diabetes. This has resulted in benign polymorphisms frequently being reported as disease-causing mutations (e.g. 38% of reported cases of *HNF1A*-MODY in Germany had benign polymorphisms [47]). Clinical selection of patients with potential monogenic diabetes is still required since, even with improved technology, next-generation sequencing cannot find a genetic aetiology in patients who have type 1 or type 2 diabetes.

## Precision medicine in type 2 diabetes: comparisons with monogenic diabetes

### Difficulty in defining aetiological subgroups in type 2 diabetes

Defining subgroups using molecular genetic testing in type 2 diabetes is very unlikely to result in discrete aetiological subgroups because the genetic predisposition is polygenic rather than monogenic and the clinical phenotype reflects environmental as well as genetic influences (Fig. 3) [48]. We can define aetiological subgroups based on physiological features, such as insulin resistance and beta cell failure. The main problems of using these categories are that these features change over time, there is a lack of agreement on optimum methods of assessment [49] and biochemical assays used in the definitions are not standardised between laboratories [50, 51]. Similar problems are seen in latent autoimmune diabetes in adults (LADA), in which there is a lack of agreement of which islet autoantibodies to study, variation in the assays used to measure a specific antibody and varying thresholds for a positive test [52]. The difficulty in defining subgroups in type 2 diabetes has a major impact on the ability to optimise treatment.

### The lack of marked differences in treatment response in type 2 diabetes

In type 2 diabetes, it is unlikely that differences in treatment will be as marked as in monogenic diabetes. On average, most glucose lowering therapies for type 2 diabetes reduce HbA_1c_ by about 1% (11 mmol/mol) [53]. It is known that there is considerable variation in treatment response to glucose lowering therapy in type 2 diabetes but, to date, there has been no description of any subgroups that respond with a dramatic 5 percentage point (31 mmol/mol) change in HbA_1c_, as observed in individuals with *HNF1A-*MODY. Pharmacogenetic impacts on treatment responses in type 2 diabetes exist but all have been small to date (<0.5% [5 mmol/mol] HbA_1c_) [54]. An alternative approach may involve defining type 2 diabetes patients who are unlikely to respond to a specific therapy, with the best example to date being insulin-treated type 2 patients with islet autoantibodies or low C-peptide who do not respond to GLP-1 receptor agonists [55].

### An alternative approach for precision medicine in type 2 diabetes

**Fig. 5:**
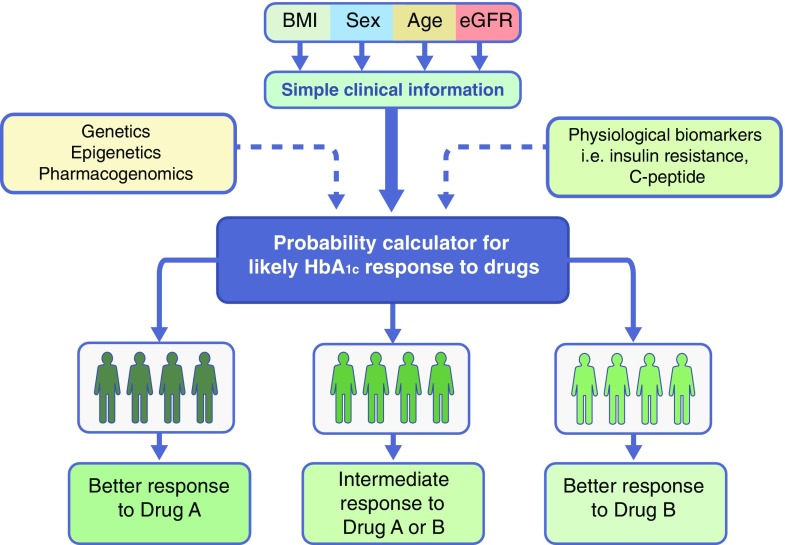
Treatment response based approach for precision diabetes in type 2 diabetes. We propose to move the focus from defining subgroups based on molecular aetiology to defining subgroups based on differential treatment response to drugs. The aim will be to create a statistical probability calculator that will use simple clinical information (e.g. age, BMI, sex, eGFR etc.) to provide a likely HbA_1c_ response to existing drugs. Large-scale routine clinical data will be used to develop a statistical model and that will be validated in already completed clinical trials. Similar approaches can also be used for a drug’s side effects. The benefits of this approach are that it will be quick to develop, easy to implement, provide clinically useful treatment choices and may incorporate future ‘omic’ or physiological biomarker discoveries

The aim of precision medicine is to find subgroups of patients that have tailored treatment. It does not specify how these subgroups are defined. The successful examples of monogenic diabetes (and also cancer) have been based on molecular genetic analysis but, as outlined above, this is unlikely to be successful in type 2 diabetes and will not be practical to bring into routine clinical practice in primary care. Therefore, rather than defining subgroups based on molecular aetiology, we suggest initial attempts to define subgroups based on differential treatment response to drugs (Fig. 5). The initial analysis for identification of this subgroup should use simple clinical data (e.g. age of diagnosis, sex and BMI) or readily available biomarkers (e.g. eGFR). This approach has the advantage that it can develop relatively quickly because of the use of already available large-scale routine clinical data and subsequently tested using clinical trial data for currently available drugs. The aim will be to create a simple calculator that will use routine clinical information to provide information on the likely HbA_1c_ response and/or risk of adverse effects for available medications. If successful, this approach will be easy to implement in clinical practice and will also be a platform on which future pharmacogenetics or ‘omic’ discoveries can add to [56]. A recent example of the successful implementation of this approach is the use of sex and BMI data for identification of patients with a preferential response to thiazolidinediones (obese female) or sulfonylureas (slim male) [57].

## Conclusions

Precision diabetes in monogenic diabetes has been an easy early win but, in contrast, its implementation in type 2 diabetes will be considerably more difficult. Monogenic diabetes has the advantage that there are discrete subgroups that are easily defined by molecular genetics. Frequently, the knowledge of the biology that results from the aetiological gene being identified has helped to define likely treatment response. These early successes have coloured our approach to precision diabetes, favouring genomics-based approaches that search for a single biomarker or a genetic variant with very large effect on treatment response. However, type 2 diabetes is a polygenic condition in which environment, as well as genetic predisposition, play a big role. In this case, an approach concentrating on newer technologies may not be optimum and certainly examining simple clinical criteria like BMI, sex and age should be carried out before rushing to molecular technologies. Finally, one thing that we have learnt from monogenic diabetes, particularly MODY, is that even when there is a clear case, both clinically and economically, for a precision diabetes approach, implementation may be difficult.

## Electronic supplementary material

Below is the link to the electronic supplementary material.

ESM 1 (PPTX 809 kb)

## Abbreviations

GLP-1: Glucagon-like peptide-1
IPEX: Immunodysregulation polyendocrinopathy enteropathy X-linked
PNDM: Permanent neonatal diabetes
TNDM: Transient neonatal diabetes
