# Supplementary material for: Precision diabetes: learning from monogenic diabetes
Source: Diabetologia. 2017 Mar 17;60(5):769–77. doi: 10.1007/s00125-017-4226-2 (PMC5907633; doi:10.1007/s00125-017-4226-2)
Supplement: Supplementary file 1 — (PPTX 809 kb) [file 125_2017_4226_MOESM1_ESM.pptx]

## Slide 1
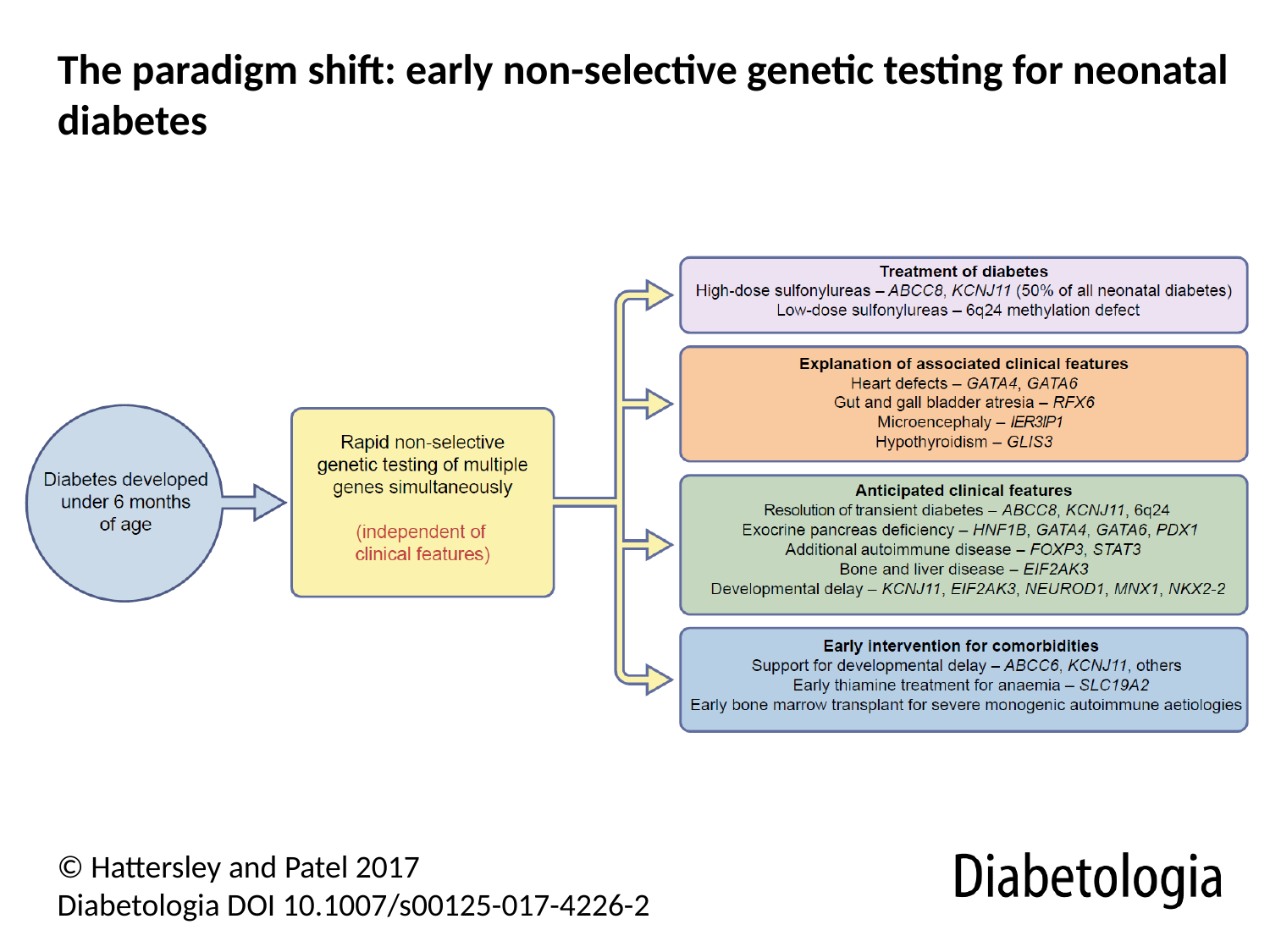

The paradigm shift: early non-selective genetic testing for neonatal diabetes
© Hattersley and Patel 2017
Diabetologia DOI 10.1007/s00125-017-4226-2

## Slide 2
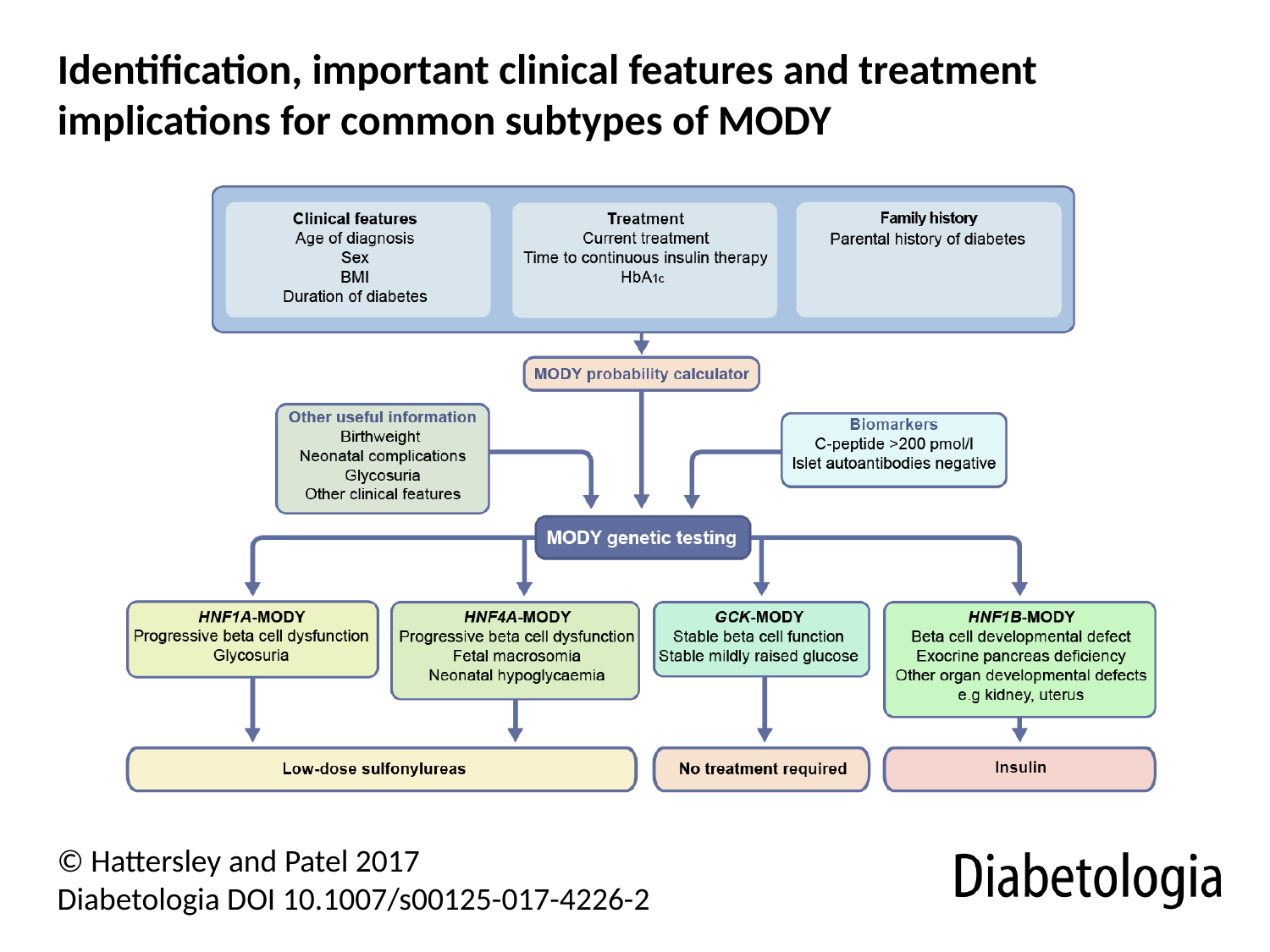

Identification, important clinical features and treatment implications for common subtypes of MODY
© Hattersley and Patel 2017
Diabetologia DOI 10.1007/s00125-017-4226-2

## Slide 3
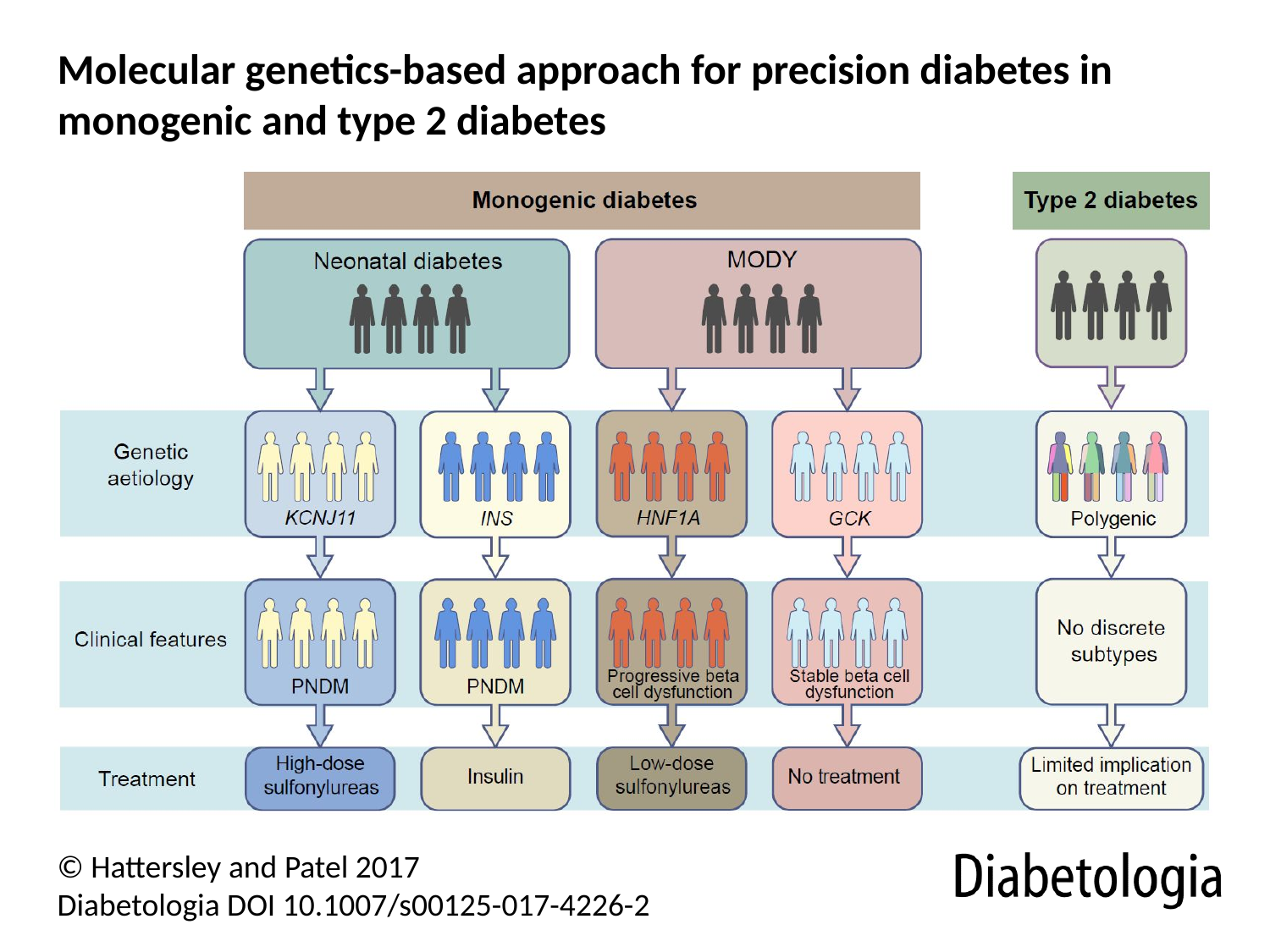

Molecular genetics-based approach for precision diabetes in monogenic and type 2 diabetes
© Hattersley and Patel 2017
Diabetologia DOI 10.1007/s00125-017-4226-2

## Slide 4
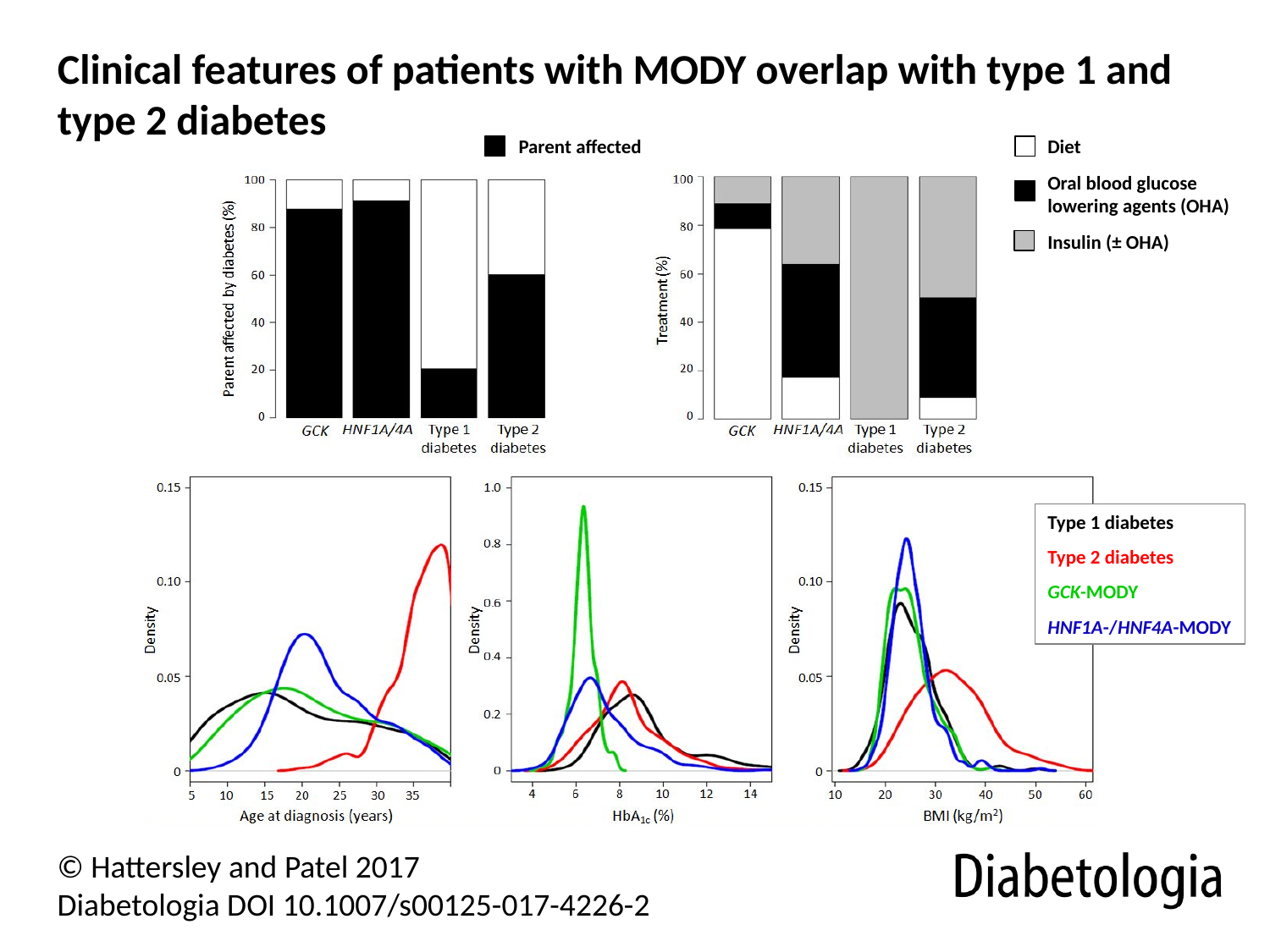

Clinical features of patients with MODY overlap with type 1 and type 2 diabetes
Diet
Oral blood glucose
lowering agents (OHA)
Insulin (± OHA)
Parent affected
Type 1 diabetes
Type 2 diabetes
GCK-MODY
HNF1A-/HNF4A-MODY
© Hattersley and Patel 2017
Diabetologia DOI 10.1007/s00125-017-4226-2

## Slide 5
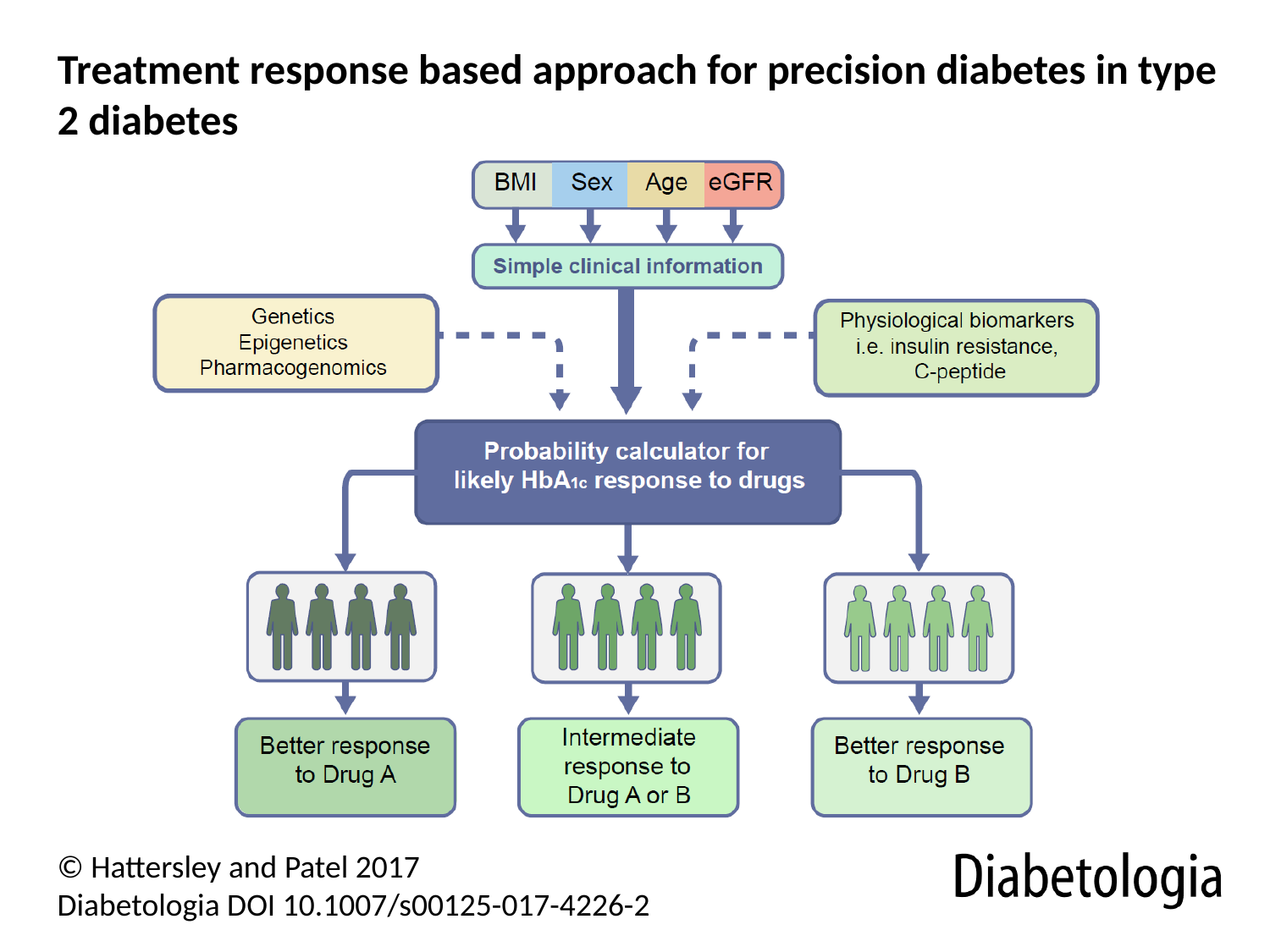

Treatment response based approach for precision diabetes in type 2 diabetes
© Hattersley and Patel 2017
Diabetologia DOI 10.1007/s00125-017-4226-2
